# Supplementary material for: Multiscale Structure of Starches Grafted with Hydrophobic Groups: A New Analytical Strategy
Source: Molecules. 2020 Jun 18;25(12):2827. doi: 10.3390/molecules25122827 (PMC7356499; doi:10.3390/molecules25122827)
Supplement: Supplementary file 1 [file molecules-25-02827-s001.pdf]

Supplementary Materials

Multiscale Structure of Starches Grafted with Hydrophobic Groups: A New Analytical Strategy

Chloé Volant [1], Alexandre Gilet [2], Fatima Beddiaf [3], Marion Collinet-Fressancourt [4,5], Xavier Falourd [3,6], Nicolas Descamps [7], Vincent Wiatz [7], Hervé Bricout [2], Sébastien Tilloy [2], Eric Monflier [2,*], Claude Quettier [7], Ahmed Mazzah [1] and Agnès Rolland-Sabaté [3,8,*]

[1] Univ. Lille, CNRS, USR3290 — MSAP — Miniaturisation pour la Synthèse, l'Analyse et la Protéomique, F-59000 Lille, France; chloe.volant@univ-ubs.fr (C.V.); ahmed.mazzah@univ-lille.fr (A.M.)
[2] Univ. Artois, CNRS, Centrale Lille, Univ. Lille, UMR 8181 – UCCS – Unité de Catalyse et Chimie du Solide, F-62300 Lens, France; alexandre.r.gilet@gmail.com (A.G.); herve.bricout@univ-artois.fr (H.B.); sebastien.tilloy@univ-artois.fr (S.T.)
[3] INRAE, UR BIA, F-44316 Nantes, France; fb.beddiaf@gmail.com (F.B.); xavier.falourd@inrae.fr (X.F.)
[4] CIRAD, UPR Recyclage et Risque, F-97743 Saint-Denis, Réunion, France; marion.collinet@cirad.fr
[5] Univ. Montpellier, Recyclage et Risque, CIRAD, 34398 Montpellier, France
[6] INRAE, BIBS facility, F-44316 Nantes, France
[7] ROQUETTE Frères, Rue de la Haute Loge, 62136 Lestrem, France; nicolas.descamps@roquette.com (N.D.); vincent.wiatz@roquette.com (V.W.); claude.quettier@roquette.com (C.Q.)
[8] INRAE, Université d'Avignon, UMR SQPOV, F-84914 Avignon, France
* Correspondence: eric.monflier@univ-artois.fr (E.M.); agnes.rolland-sabate@inrae.fr (A.R.-S.); Tel.: +33-(0)3-2179-1772 (E.M.); +33-(0)4-3272-2522 (A.R.-S.)

S1. Solubility of esterified and etherified starches

The solubility of acetylated starches in various solvents was evaluated after stirring during 5 days in the solvent at room temperature at a concentration of 0.5 g L^{-1}. It was evaluated by transmittance (%T) and dynamic light scattering (DLS) measurements. The tested solvents were dimethylsulfoxide (DMSO), tetrahydrofuran (THF), acetonitrile (ACN) and water. Light transmittance (%T) was determined at 650 nm against a solvent blank with a Jasco V-530 spectrophotometer (Jasco Corporation, Tokyo, Japan) and the size (hydrodynamic radius, R_H) of particles and aggregates was determined by DLS with a Malvern Zetasizer Nano ZS90 Instrument (Malvern, UK).

Table S1a. Solubility of acetylated starches

**Reference**	**Solvent**	**Transmittance (% T, 650 nm)**	**$\overline{R}_H$ (nm)**	**Visible particles**
AWMS	DMSO	99.9	198	-
	THF	96.4	123	+
	ACN	89.0	124	++
	Water	<50	NA	+++
APOS	DMSO	99.9	186	-
	THF	99.1	377	+
	ACN	NA	NA	+
	Water	<50	NA	+++
APES	DMSO	99.5	240	-
	THF	92.6	16	+
	ACN	97.7	173	+
	Water	<50	NA	+++

NA : Not available.

All the acetylated starches were soluble in pure DMSO (%T > 99 % and hydrodynamic radius were around 200 nm (Table S1a and [1,2]). They were not completely soluble in THF and ACN (%T around 90 % and visible insoluble particles) and they were insoluble in water (%T <50, many visible particles). DLS measurements had to be taken with care as the molecular size of starches were too high to be determined with DLS at one angle only [3], by consequence they have to be considered only as a cross-check with the transmittance measurements.

Table S1b. Solubility of etherified starches

Reference	Solvent	Visible particles
HDo-POS-1	DMSO	-
	THF	-
	$CDCl_3$	-
	ACN	++
	MeOH	++
	Water	++
HDo-POS-2	DMSO	-
	THF	-
	$CDCl_3$	-
	ACN	++
	MeOH	++
	Water	++
HDo-HPhe-POS-1	DMSO	+
	THF	+
	$CDCl_3$	-
	ACN	++
	MeOH	++
	Water	++
HDo-HPhe-POS-2	DMSO	+
	THF	-
	$CDCl_3$	-
	ACN	++
	MeOH	++
	Water	++

Visual control showed that all the etherified starches were soluble in organic solvents, i.e. in DMSO for HDo-POS-1 and HDo-POS-2, $CDCl_3$ for HDo-HPhe-POS-1, THF and $CDCl_3$ for HDo-HPhe-POS-2.

References

1. Rolland-Sabaté, A.; Colonna, P.; Mendez-Montealvo, M.G.; Planchot, V. Branching Features of Amylopectins and Glycogen Determined by Asymmetrical Flow Field Flow Fractionation

Coupled with Multiangle Laser Light Scattering. *Biomacromolecules* **2007**, *8*, 2520-2532, doi:10.1021/bm070024z.

2. Rolland-Sabaté, A.; Guilois, S.; Jaillais, B.; Colonna, P. Molecular size and mass distributions of native starches using complementary separation methods: Asymmetrical Flow Field Flow Fractionation (A4F) and Hydrodynamic and Size Exclusion Chromatography (HDC-SEC). *Analytical and Bioanalytical Chemistry* **2011**, *399*, 1493-1505, doi:10.1007/s00216-010-4208-4.
3. Roger, P.; Bello-Perez, L.A.; Colonna, P. Contribution of amylose and amylopectin to the light scattering behaviour of starches in aqueous solution. *Polymer* **1999**, *40*, 6897-6909, doi:http://dx.doi.org/10.1016/S0032-3861(99)00051-8.

S2. The complete deacetylation was checked by Fourier Transformed Infrared Spectroscopy (FTIR)

The infrared absorption spectra of tablets constituted by 2 mg of sample and 120 mg of KBr were obtained with a resolution of 1 cm^{-1} in the 700–4000 cm^{-1} wave number range, using a Fourier Transform Infrared (FTIR) Spectrometer (Tensor 27, BRUKER) equipped with an Attenuated Total Reflection system (ATR, PIKE).

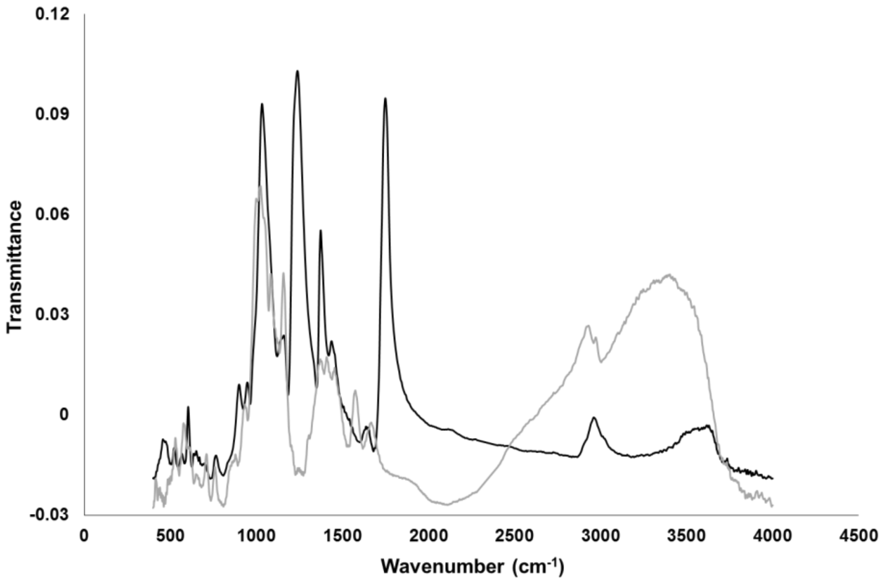

Figure S2. FTIR spectra of AWMS and deacetylated AWMS

Acetylated WMS, AWMS (black) and deacetylated AWMS (grey)

The bands characteristic to acetyl groups at 1731 and 1249 cm^{-1} [4] disappeared completely during the deacetylation process.

References

4. Sun, S.; Zhang, G.; Ma, C. Preparation, physicochemical characterization and application of acetylated lotus rhizome starches. *Carbohydrate Polymers* **2016**, *135*, 10-17, doi:https://doi.org/10.1016/j.carbpol.2015.07.090.

S3. Chain length distribution of acetylated starches

Table S3. Chain length distribution of debranched native and acetylated starches obtained from HPAEC-PAD.

Type of Starch	Peak DP		% distribution					Average CL	Highest detectable DP
	I	II	DP 6-9	DP 6-12	DP 13-24	DP 25-36	DP≥37		
WMS	12	ND	7.8 (0.01)	28.4 (0.32)	53.3 (0.39)	12.3 (0.11)	6.0 (0. 59)	23.0 (0.32)	86
AWMS	11	ND	14.0 (0.19)	37.5 (0.09)	46.5 (0.15)	9.4 (0.15)	6.5 (0.22)	20.6 (0.12)	94
POS	12	49	9.8 (0.06)	25.0 (0.09)	44.3 (0.04)	12.4 (0.01)	18.3 (0.03)	23.5 (0.03)	106
APOS	12	47	11.6 (NA)	29.2 (NA)	45.8 (NA)	10.6 (NA)	14.4 (NA)	ND	81
PES	12	45	8.0 (0.01)	23.6 (0.22)	46.8 (0.372)	14.3 (0.06)	15.3 (0.53)	22.9 (0.29)	105
APES	12	44	8.8 (0.06)	25.3 (0.23)	47.6 (0.33)	13.8 (0.01)	13.3 (0.64)	21.8 (0.38)	96

Standard deviations are given in parenthesis; NA: Not available; ND : Not detected; CL: Chain length.

S4. Surface composition of acetylated and etherified starches studied by TOF-SIMS

TOF-SIMS is a non-quantitative method for surface analysis but provides data on surface chemical composition at the micrometer scale (500 µm², 128×128 pixel, depth: 1-2 nm). A higher resolution was observed for film samples than for the corresponding powdered sample, the plane surface favoring the focus of the ion beam.

On the positive spectrum of WMS powder (Figure S4a), surface exhibits some peaks assigned to anhydroglucose unit, when supplementary ions were detected on AWMS spectrum (Table S4a). APOS and APES surface showed the same peaks in positive mode.

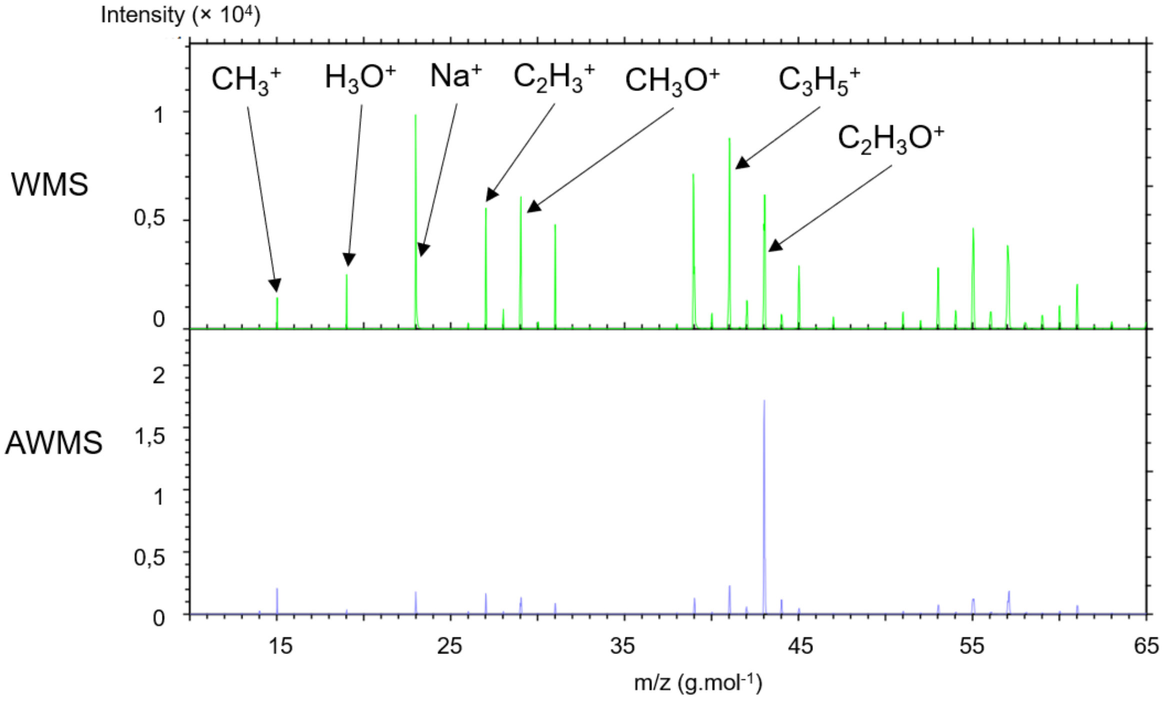

Figure S4a. TOF-SIMS spectra of a WMS and AWMS in positive mode (10-65 g mol^{-1})

Table S4a. Fragment ions identified on WMS, POS, PES, AWMS, APOS and APES spectra on positive and negative mode

	Ion	Attribution	Identification
Positive mode	23	Na^+	Anhydroglucose unit
	27	$C_2H_3^+$	Anhydroglucose unit
	31	CH_3O^+	Anhydroglucose unit
	41	$C_3H_5^+$	Anhydroglucose unit
	43	$C_2H_3O^+$	Acetate group
	69	$C_4H_5O^+$	Anhydroglucose unit
	81	$C_5H_5O^+$	Acetate group
	85	$C_4H_5O_2^+$	Anhydroglucose unit
	97	$C_5H_5O_2^+$	Acetate group
	109	$C_6H_5O_2^+$	Acetate group
Negative mode	45	C_2HO^-	Anhydroglucose unit
	58	$C_2H_2O_2^-$	Acetate group
	59	$C_2H_3O_2^-$	Acetate group
	63	PO_2^-	Anhydroglucose unit
	79	PO_3^-	Anhydroglucose unit

The most intense ion is the acetate fragment, which is distributed homogeneously for AWMS, APOS and APES. No remaining granules are observed for AWMS, APOS and APES surfaces.

The elemental composition of the film surface of the ether prototypes was also investigated by TOF-SIMS in the same conditions than those developed for model starches.

HDo-POS-1 and HDo-POS-2 surface of film were analyzed in positive polarity (Figure S4b). Spectra displayed fragments of starch in addition to fragments of fatty chains. Elemental composition is identical for the 2 prototypes with detection of alkyl fragments and fragments of hydroxyethers groups (Table S4b). TOF-SIMS is not a quantitative method, however the relative intensities of the spectra indicate that ions are better detected on film surface, acquisition being facilitated on its flat surface. The shaping could also influence the organization of the material and promote a phenomenon of exudation of the epoxydodecane derivatives.

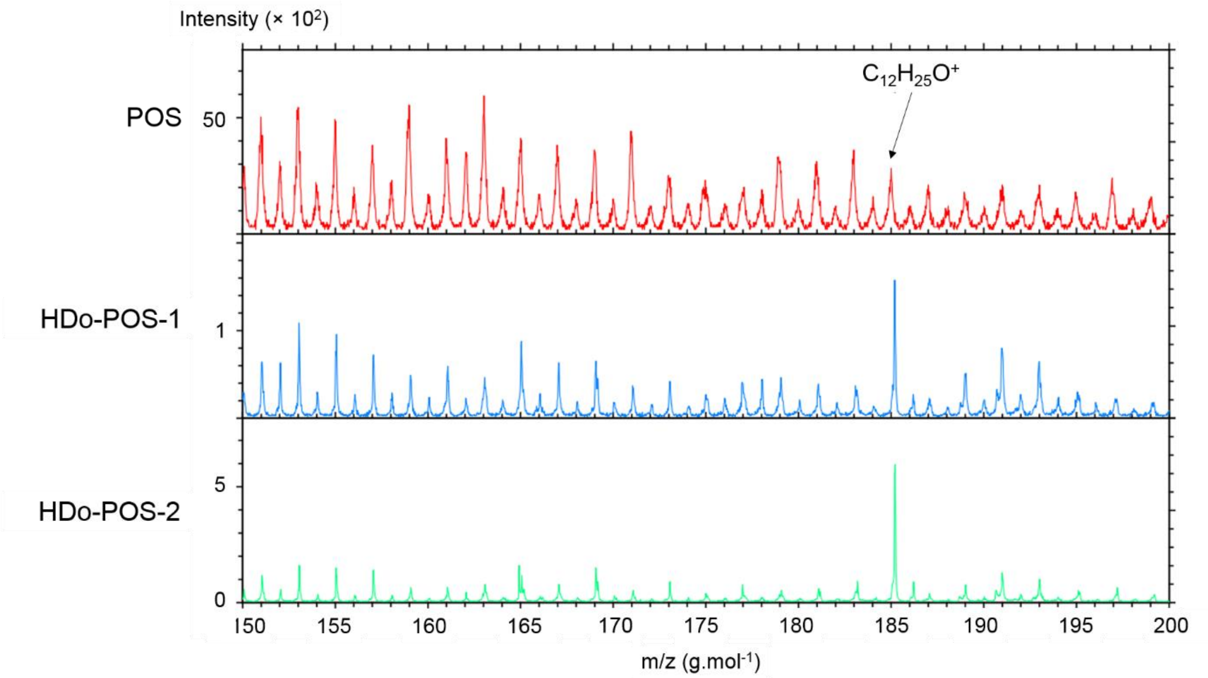

Figure S4b. TOF-SIMS spectra of a POS, HDo-POS-1 and HDo-POS-2 in positive mode (150 to 200 g mol^{-1})

In negative polarity, fragments originating from starch and epoxydodecane are identified in HDo-POS-1 and HDo-POS-2 (Figure S4b and Table S4b).

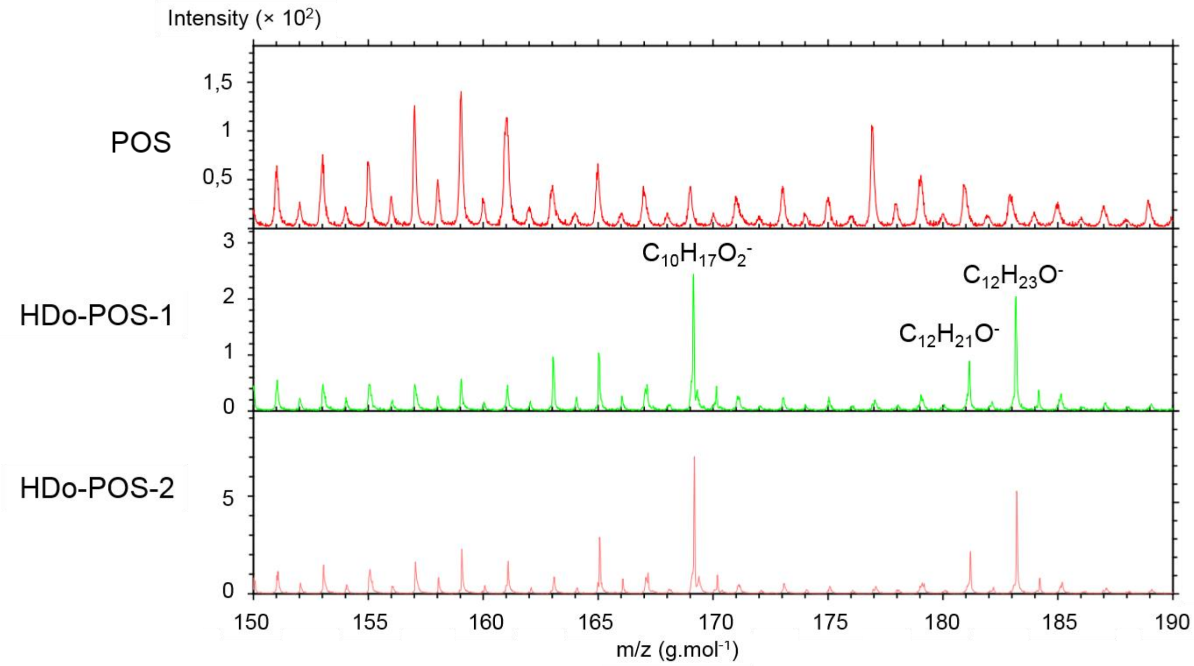

Figure S4c. TOF-SIMS spectra of a POS, HDo-POS-1 and HDo-POS-2 in negative mode (150 and 190 g mol^{-1})

Table S4b. Fragment ions identified on HDo-POS-1 and HDo-POS-2 spectra on positive and negative mode

	Ion	Attribution	Identification
Positive mode	127	$C_6H_7O_3^+$	Starch
	185	$C_{12}H_{25}O^+$	Starch
	385	$C_{24}H_{49}O_3^+$	Starch
Negative mode	59	$C_2H_3O_2^-$	Starch
	71	$C_4H_7O^-$	Starch
	87	$C_4H_7O_2^-$	Starch
	101	$C_6H_{13}O^-$	Starch
	113	$C_7H_{13}O^-$	Starch
	141	$C_9H_{17}O^-$	Starch
	169	$C_{10}H_{17}O_2^-$	Epoxydodecane
	181	$C_{12}H_{21}O^-$	Epoxydodecane
	183	$C_{12}H_{23}O^-$	Epoxydodecane

In positive polarity (Figures S4d and S4e), spectra of HDo-HPhe-POS-1 and HDo-HPhe-POS-2 showed fragments from hydroxyethers and phenyl (Table S4c). Although TOF-SIMS is not quantitative, we noticed a low intensity for aromatic fragments, this may be due to the small quantity introduced into the prototypes. Fragments from starch are not detected, probably because of its small amount on surface.

We identified starch fragments in negative polarity for HDo-HPhe-POS-1 and HDo-HPhe-POS-2 (Table S4c).

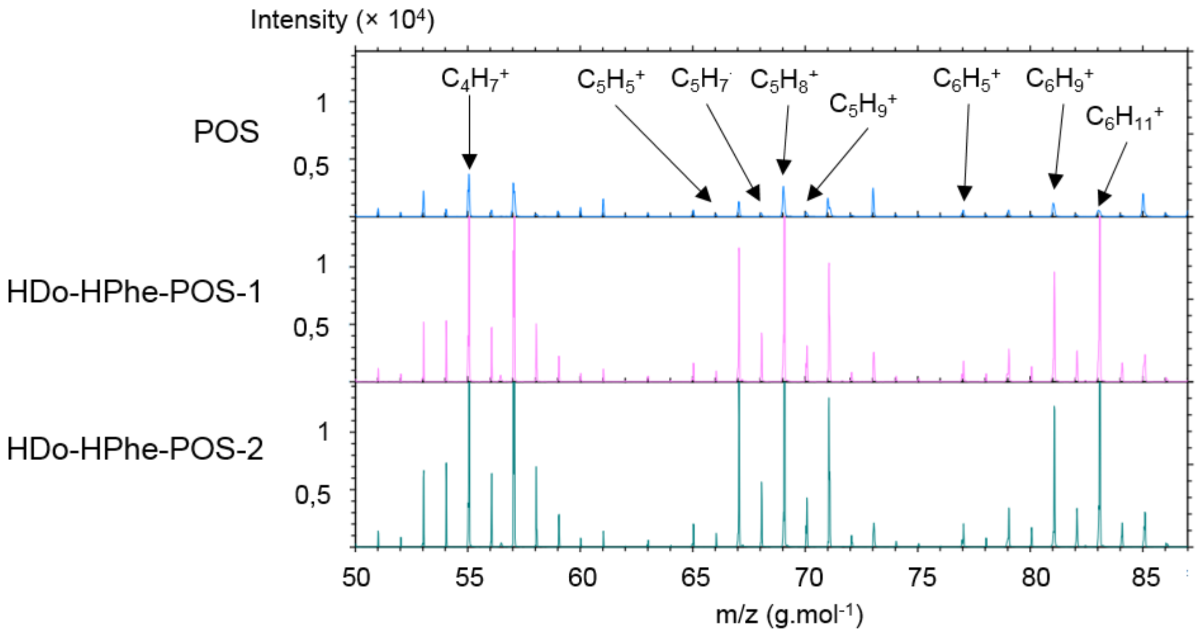

Figure S4d. TOF-SIMS spectra of a POS, HDo-HPhe-POS-1 and HDo-HPhe-POS-2 in positive mode (50 and 85 g mol^{-1})

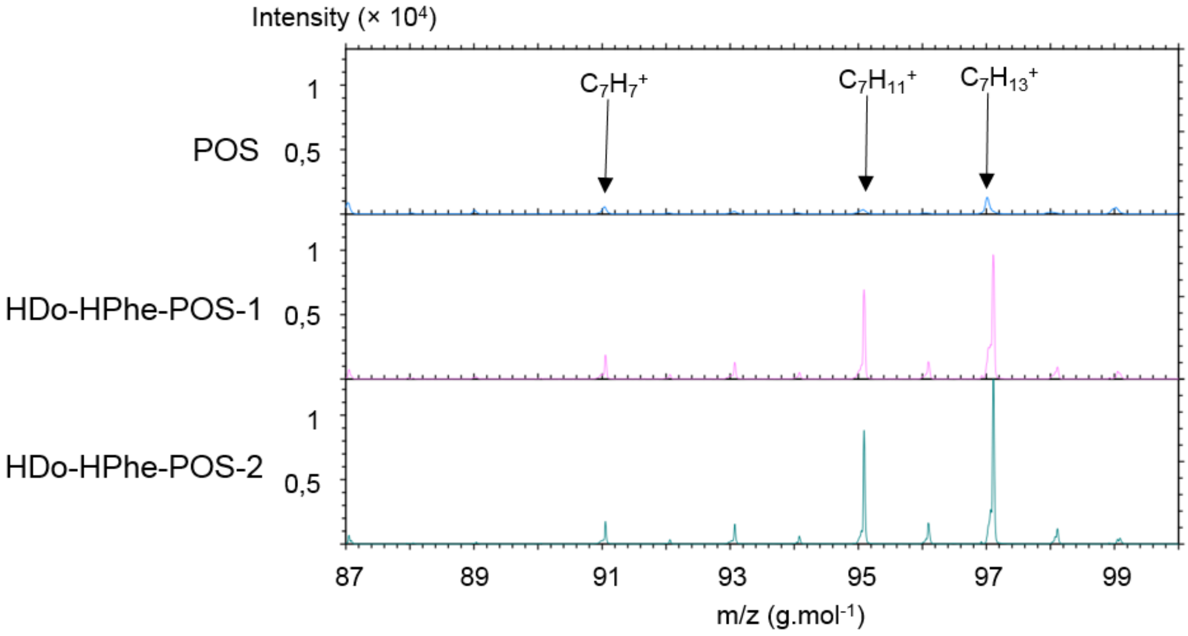

Figure S4e. TOF-SIMS spectra of a POS, HDo-HPhe-POS-1 and HDo-HPhe-POS-2 in positive mode (87 and 100 g mol^{-1})

Table S4c. Fragment ions identified on HDo-HPhe-POS-1 and HDo-HPhe-POS-2 spectra on positive and negative mode

	Ion	Attribution	Identification
Positive mode	91	$C_7H_7^+$	Phenyl
	97	$C_7H_{11}^+$	Phenyl
	99	$C_7H_{13}^+$	Phenyl
	185	$C_{12}H_{25}O^+$	Hydroxyether
	385	$C_{24}H_{49}O_3^+$	Hydroxyether
	773	$C_{48}H_{101}O_6^+$	Hydroxyether
Negative mode	59	$C_2H_3O_2^-$	Starch
	71	$C_4H_7O^-$	Starch
	87	$C_4H_7O_2^-$	Starch

Surface ion repartition was studied by TOF-SIMS imaging on three distinct areas. Epoxydodecane fragment $C_{12}H_{25}O^+$ seems uniformly distributed on HDO-POS-1 and HDO-POS-2 films (Figure S4f).

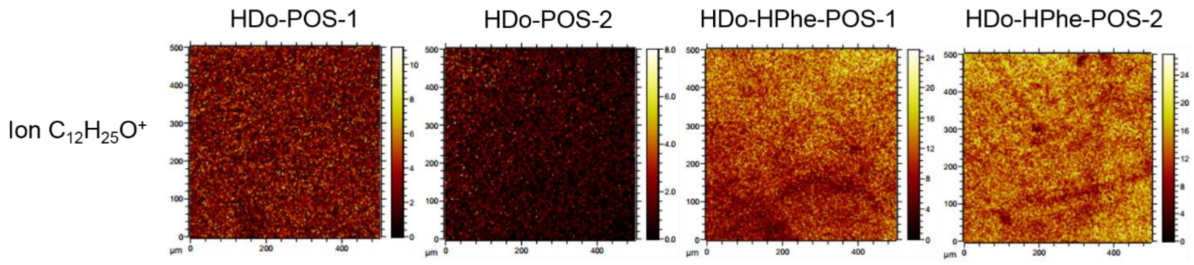

Figure S4f. TOF-SIMS imaging of HDo-POS-1, HDo-POS-2, HDo-HPhe-POS-1 and HDo-HPhe-POS-2 for the epoxydodecane major fragment

HDo-HPhe-POS-1 and HDo-HPhe-POS-2 contain a phenyl derivative in different proportion, it is possible to control the distribution of fragments of this derivative (Figure S4g).

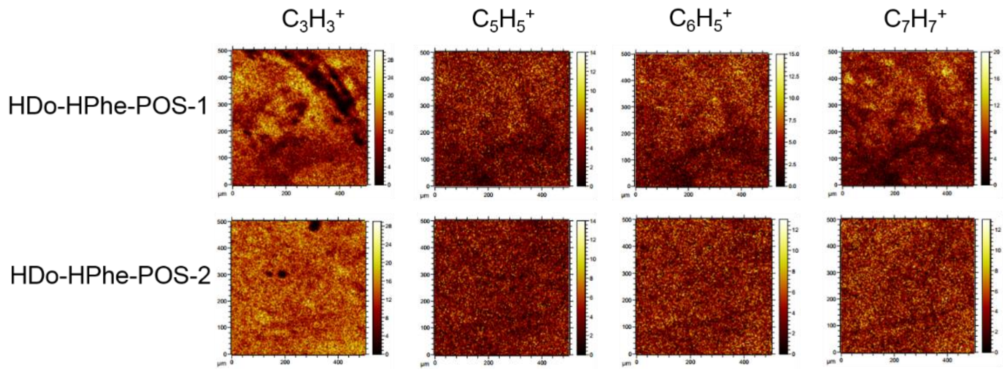

Figure S4g. TOF-SIMS imaging of HDo-HPhe-POS-1 and HDo-HPhe-POS-2 for the epoxyphenylether major fragments

S5. Determination of the degree of substitution (DS) of the 2-hydroxydodecyl potato starches (HDo-POS-1 and HDo-POS-2) by elemental analysis (EA)

1) Demonstration of the $\mathbf{DS_{HDo.EA(C)}}$ expression (DS deducted from the %C)

The expression of $DS_{HDo.EA(C)}$, i.e. the number of 2-hydroxydodecyl groups (HDo) per AGU determined by elemental analysis from wt% of C (%C) in the 2-hydroxydodecyl starch, can be easily demonstrated:

$$\%C = \frac{\text{Mass of C in a modified AGU (g mol}^{-1})}{\text{Mass of a modified AGU (g mol}^{-1})} \times 100$$

$$\%C = \frac{\text{Mass of C in an unmodified AGU } + \text{Mass of C due to the HDo graft presence}}{\text{Mass of a unmodified AGU } + \text{Mass coming from the HDo graft presence}} \times 100$$

Where masses are in g mol^{-1}.

$$\%C = \frac{6\ M_C + DS \times 12\ M_C}{M_{AGU} + DS \times (M_{HDo} - M_H)} \times 100$$

M_C, M_H, M_{AGU} and M_{HDo} = Molar mass (g mol^{-1}) of C, H, AGU ($C_6H_{10}O_5$) and 2-hydroxydodecyl graft ($C_{12}H_{25}O$)

M_C = 12.010736 g mol^{-1}

M_H = 1.007941 g mol^{-1}

M_{AGU} = 162.140600 g mol^{-1}

M_{HDo} = 185.326300 g mol^{-1}

$$\%C = \frac{600\ M_C + DS\ \times 1200\ M_C}{M_{AGU} + DS \times (M_{HDo} - M_H)}$$

$$DS \times [(M_{HDo} - M_H) \times\ \%C\ -\ 1200\ M_C] = 600\ M_C - \%C \times M_{AGU}$$

finally,

$$DS_{HDo.EA(C)} = \frac{\%C \times M_{AGU} - 600\ M_C}{1\ 200\ M_C - \%C \times (M_{HDo} - M_H)}$$

2) Demonstration of the $\mathbf{DS_{HDo.EA(H)}}$ expression (DS deducted from the %H)

The expression of $DS_{HDo.EA(H)}$, i.e. the number of 2-hydroxydodecyl groups (HDo) per AGU determined by elemental analysis from wt% of H (%H) in the 2-hydroxydodecyl starch, can be also easily demonstrated:

$$\%H = \frac{\text{Mass of H in a modified AGU (g mol}^{-1})}{\text{Mass of a modified AGU (g mol}^{-1})} \times 100$$

$$\%H = \frac{\text{Mass of H in an unmodified AGU } + \text{Mass of H due to the HDo graft presence}}{\text{Mass of a unmodified AGU } + \text{Mass coming from the HDo graft presence}} \times 100$$

Where masses are in g mol^{-1}.

$$\%H = \frac{10\ M_H + DS \times 24\ M_H}{M_{AGU} + DS \times (M_{HDo} - M_H)} \times 100$$

$$\%H = \frac{1000\ M_H + DS \times 2400\ M_H}{M_{AGU} + DS \times (M_{HDo} - M_H)}$$

$$DS \times [(M_{HDo} - M_H) \times \ \%H \ - \ 2400\ M_H] = 1000\ M_H - \%H \times M_{AGU}$$

and finally,

$$\boxed{DS_{HDo.EA(H)} = \frac{\%H \times M_{AGU} - 1\ 000\ M_H}{2\ 400\ M_H - \%H \times (M_{HDo} - M_H)}}$$

The DS was determined by elemental analysis (EA) from %C ($DS_{HDo.EA(C)}$) and from %H ($DS_{HDo.EA(H)}$) by using the following formulas:

$$DS_{HDo.EA(C)} = \frac{\%C \times M_{AGU} - 600\ M_C}{1\ 200\ M_C - \%C \times (M_{HDo} - M_H)}$$

$$DS_{HDo.EA(H)} = \frac{\%H \times M_{AGU} - 1\ 000\ M_H}{2\ 400\ M_H - \%H \times (M_{HDo} - M_H)}$$

Where M_{AGU}, M_C, M_H and M_{HDo} are the molar mass of AGU ($C_6H_{10}O_5$), C, H and 2-hydroxydodecyl graft ($C_{12}H_{25}O$) (in g mol^{-1}) and %C and %H the wt% of carbon and hydrogen in the product, determined by elemental analysis (%).

S6. Determination of the degree of substitution (DS) of HDo-POS-1 by ^{1}H-NMR analysis

The $DS_{HDo.lNMR}$, i.e the average number of 2-hydroxydodecyl grafts per AGU, was determined by ^{1}H-NMR analysis of the product dissolved in DMSO-d6, by using the following formulas based on signals corresponding to the aliphatic part of the graft, compared to the signal of the anomeric proton:

$$DS_{HDo.NMR} = \frac{I_{Me}}{3\ I_{H1}} \text{ or } \frac{I_{Dec}}{21\ I_{H1}}$$

I_{Me}= Integration of the methyl NMR signal (0.75-0.9 ppm)

I_{Dec}= Integration of the decyl (-$(CH_2)_9CH_3$)) NMR signal of 2-hydroxydodecyl grafts (0.75-1.5 ppm)

I_{H1}= Integration of the anomeric proton NMR signal (4.8-5.4 ppm)

S7. Determination of the degree of substitution (DS) of the 2-hydroxydodecyl 2-hydroxyphenethyl potato starches (HDo-HPhe-POS-1 and HDo-HPhe-POS-2) by ^{1}H-NMR analysis

The substitution degrees corresponding to 2-hydroxydodecyl/2-hydroxyphenethyl groups were determined by analyzing the ^{1}H-NMR spectra of the two mixed starch ethers in $CDCl_3$ and in THF-d_8. Whatever the solvent used ($CDCl_3$ or THF-d_8), three principles regions could be distinguished on each spectrum (Fig. S8):

- the **0.7-1.6 ppm region** contains the decyl moiety signal (**21 H**)
- the **3.0-6.0 ppm region** contains H_1, H_2, H_3, H_4, H_5, H_6 protons signal of the AGU (**7 H**) and overlapped signals of CH_2 and CH groups (**3 H**) of the O-CH_2-CH(OH) moiety of the two grafts types (in the spectrum done in THF-d8, this part of the spectrum was overlapped by the CH_2-O signal of residual THF at 3.76 ppm)
- the **7.0-7.5 ppm region** contains the phenyl signal (**5 H**) (in the spectrum done in $CDCl_3$, the phenyl signal was overlapped by the signal of residual $CHCl_3$ at 7.26 ppm).

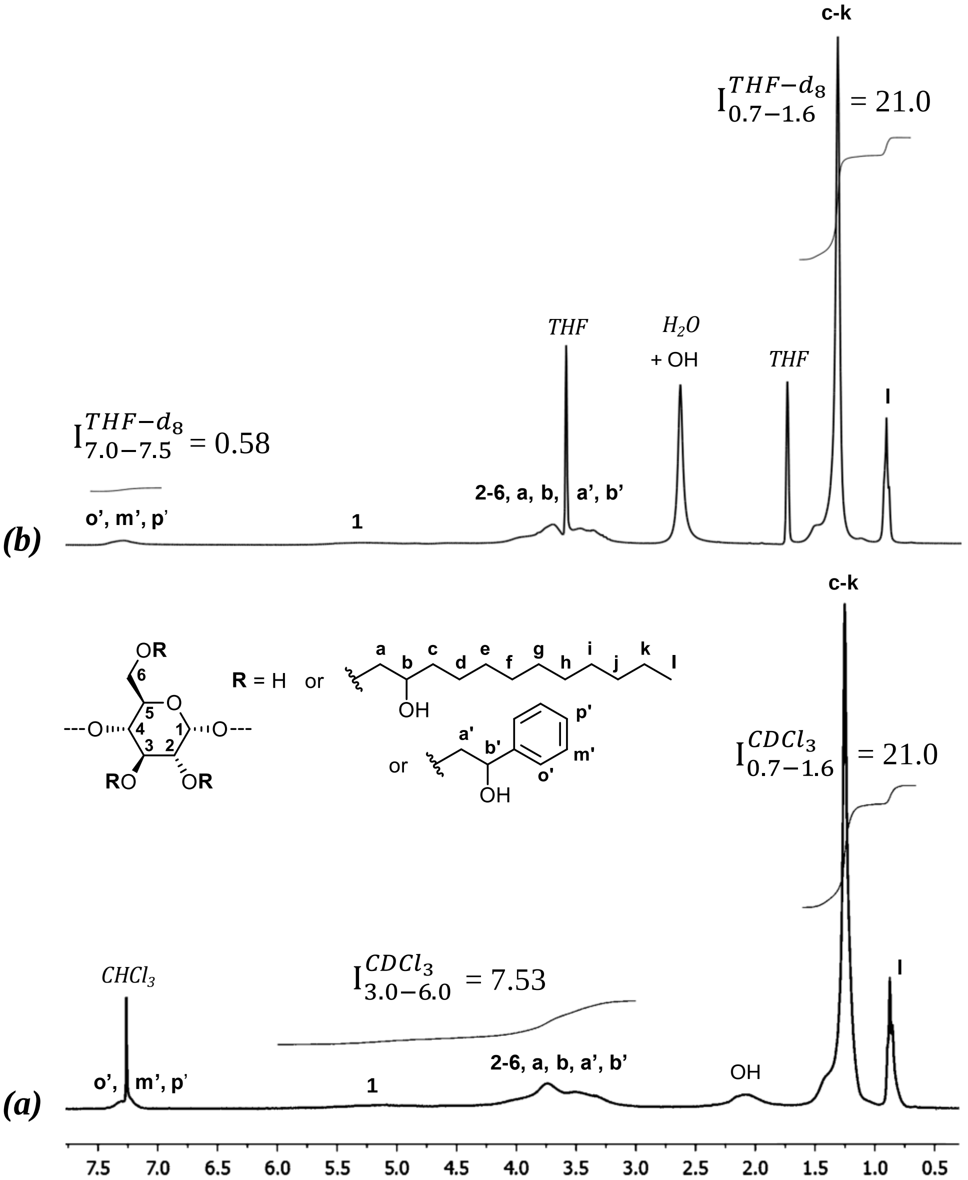

Figure S7. ^{1}H-NMR spectra of a 2-hydroxydodecyl 2-hydroxyphenethyl potato starch (here, HDo-HPhe-POS-2)

(a) in $CDCl_3$ and (b) in THF-d_8 (300 MHz, 25 °C)

The DS for each graft type ($DS_{HDo.NMR}$ and $DS_{HPhe.NMR}$, defined as the number of 2-hydroxydodecyl (HDo) and 2-hydroxyphenethyl (HPhe) groups per AGU determined by ^{1}H-NMR) could be defined by the two following formulas:

$$DS_{HDo.NMR} = \frac{I_{one\ H\ of\ Dec}}{I_{one\ H\ of\ AGU}} \qquad DS_{HPhe.NMR} = \frac{I_{one\ H\ of\ Ph}}{I_{one\ H\ of\ AGU}}$$

where $I_{one\ H\ of\ Dec}$, $I_{one\ H\ of\ Ph}$ and $I_{one\ H\ of\ AGU}$ represent the intensity of one H of the decyl moiety, of the phenyl moiety and of the AGU moiety, on the same ^{1}H-NMR spectrum.

So, in the absence of solvent residual signals ($CHCl_3$ and THF) and by naming $I_{X\text{-}Y}$ the intensity of the ^{1}H-NMR signal one the same spectrum between X and Y ppm, the two previous formulas become:

$$DS_{HDo.NMR} = \frac{I_{one\ H\ of\ Dec}}{I_{one\ H\ of\ AGU}} = \frac{\frac{I_{0.7-1.6}}{21}}{\frac{I_{3.0-6.0} - \frac{3}{21} \times I_{0.7-1.6} - \frac{3}{5} \times I_{7.0-7.5}}{7}}$$

$$DS_{HPhe.NMR} = \frac{I_{one\ H\ of\ Ph}}{I_{one\ H\ of\ AGU}} = \frac{\frac{I_{7.0-7.5}}{5}}{\frac{I_{3.0-6.0} - \frac{3}{21} \times I_{0.7-1.6} - \frac{3}{5} \times I_{7.0-7.5}}{7}}$$

and, after simplification,

$$DS_{HDo.NMR} = \frac{35 \times I_{0.7-1.6}}{105 \times I_{3.0-6.0} - 15 \times I_{0.7-1.6} - 63 \times I_{7.0-7.5}}$$

$$DS_{HPhe.NMR} = \frac{49 \times I_{7.0-7.5}}{35 \times I_{3.0-6.0} - 5 \times I_{0.7-1.6} - 21 \times I_{7.0-7.5}}$$

The $I_{0.7-1.6}$ and $I_{3.0-6.0}$ integrations were measured on the spectrum done in $CDCl_3$ and could therefore be written as $I_{0.7-1.6}^{CDCl_3}$ and $I_{3.0-6.0}^{CDCl_3}$, respectively.

Because in $CDCl_3$, the phenyl signal was overlapped by the signal of residual $CHCl_3$ at 7.26 ppm, the value $I_{7.0-7.5}^{CDCl_3}$ did not only represent the phenyl protons (indeed, $I_{7.0-7.5}^{CDCl_3} > I_{Phe}^{CDCl_3}$, with $I_{Phe}^{CDCl_3}$ = intensity of the phenyl group in $CDCl_3$). However, $I_{Phe}^{CDCl_3}$ could be deduced from the following equation, because the ratio between two signals of a ^{1}H-NMR spectrum is independent of the solvent in which this spectrum is recorded.

$$R = \frac{I_{Phe}^{CDCl_3}}{I_{Dec}^{CDCl_3}} = \frac{I_{Phe}^{THF-d_8}}{I_{Dec}^{THF-d_8}}$$

where $I_{Phe}^{CDCl_3}$, $I_{Phe}^{THF-d_8}$, $I_{Dec}^{CDCl_3}$ and $I_{Dec}^{THF-d_8}$, the intensities of the phenyl group and of the decyl group in the ^{1}H-NMR spectra done in $CDCl_3$ and THF, respectively. So,

$$I_{Phe}^{CDCl_3} = R \times I_{Dec}^{CDCl_3}$$

and, more precisely $\quad I_{Phe}^{CDCl_3} = R \times I_{0.7-1.6}^{CDCl_3} \quad$ with $\quad R = \frac{I_{7.0-7.5}^{THF-d_8}}{I_{0.7-1.6}^{THF-d_8}}$

By replacing $I_{7.0-7.5}$ by $I_{Phe}^{CDCl_3} (= R \times I_{0.7-1.6}^{CDCl_3})$ in the previous expressions of $DS_{HDo.NMR}$ and $DS_{HPhe.NMR}$, we obtained the following expressions.

$$DS_{HDo.NMR} = \frac{35 \times I_{0.7-1.6}^{CDCl_3}}{105 \times I_{3.0-6.0}^{CDCl_3} - 15 \times I_{0.7-1.6}^{CDCl_3} - 63 \times R \times I_{0.7-1.6}^{CDCl_3}}$$

$$DS_{HPhe.NMR} = \frac{49 \times R \times I_{0.7-1.6}^{CDCl_3}}{35 \times I_{3.0-6.0}^{CDCl_3} - 5 \times I_{0.7-1.6}^{CDCl_3} - 21 \times R \times I_{0.7-1.6}^{CDCl_3}}$$

and finally,

$$DS_{HDo.NMR} = \frac{35 \times I_{0.7-1.6}^{CDCl_3}}{105 \times I_{3.0-6.0}^{CDCl_3} - (15 + 63\ R) \times I_{0.7-1.6}^{CDCl_3}} \qquad \text{with} \qquad R = \frac{I_{7.0-7.5}^{THF-d_8}}{I_{0.7-1.6}^{THF-d_8}}$$

$$DS_{HPhe.NMR} = \frac{49 \times R \times I_{0.7-1.6}^{CDCl_3}}{35 \times I_{3.0-6.0}^{CDCl_3} - (5 + 21\ R) \times I_{0.7-1.6}^{CDCl_3}}$$

$DS_{HDo.NMR}$ = number of 2-hydroxydodecyl groups (HDo) per AGU determined by ^{1}H-NMR

$DS_{HPhe.NMR}$ = number of 2-hydroxyphenethyl groups (HPhe) per AGU determined by ^{1}H-NMR

$I_{0.7-1.6}^{CDCl_3}$ = integration of the ^{1}H-NMR spectrum done in $CDCl_3$ between 0.7 and 1.6 ppm (decyl part of the HDo group (21H))

$I_{3.0-6.0}^{CDCl_3}$ = integration of the ^{1}H-NMR spectrum done in $CDCl_3$ between 3.0 and 6.0 ppm (H_1, H_2, H_3, H_4, H_5, H_6 protons signal of the AGU (7H) and overlapped signals of CH_2 and CH groups (3H) of the O-CH_2-CH(OH) moiety of the two grafts types (in the spectrum done in THF-d_8, this part of the spectrum was overlapped by the CH_2-O signal of residual THF at 3.76 ppm).

R = $I_{7.0-7.5}^{THF-d_8}$ / $I_{0.7-1.6}^{THF-d_8}$ = ratio between the integrations of the ^{1}H-NMR spectrum between 7.0 and 7.5 ppm (phenyl signal) and between 0.7 and 1.6 ppm (decyl signal) of the compound dissolved in THF-d_8 (in the spectrum done in $CDCl_3$, the phenyl signal was overlapped by the signal of residual $CHCl_3$ at 7.26 ppm).

As an example, using the values of the integrations of **HDo-HPhe-POS-2** ^{1}H-NMR spectra done in $CDCl_3$ and THF-d_8 (cf. Figure S8: $I_{0.7-1.6}^{CDCl_3}$ = 21.0, $I_{3.0-6.0}^{CDCl_3}$ = 7.53, $I_{0.7-1.6}^{THF-d_8}$ = 21.0 and $I_{7.0-7.5}^{THF-d_8}$ = 0.58), the above formulas gave R = 0.0276 and, after calculation, $DS_{HDo.NMR} =$ 1.67 and $DS_{HPhe.NMR} =$ 0.19.

For **HDo-HPhe-POS-1**, and with the same method, we determined $DS_{HDo.NMR} =$ 1.45 and $DS_{HPhe.NMR} =$ 0.10.
